# Dysregulated early transcriptional signatures linked to mast cell and interferon responses are implicated in COVID-19 severity

**DOI:** 10.3389/fimmu.2023.1166574

**Published:** 2023-05-16

**Authors:** Rachel MacCann, Alejandro Abner Garcia Leon, Gabriel Gonzalez, Michael J. Carr, Eoin R. Feeney, Obada Yousif, Aoife G. Cotter, Eoghan de Barra, Corinna Sadlier, Peter Doran, Patrick W. Mallon

**Affiliations:** ^1^ School of Medicine, University College Dublin, Dublin, Ireland; ^2^ Department of Infectious Diseases, St. Vincent’s University Hospital, Dublin, Ireland; ^3^ Centre for Experimental Pathogen Host Research (CEPHR), University College Dublin, Dublin, Ireland; ^4^ National Virus Reference Laboratory, University College Dublin, Dublin, Ireland; ^5^ Japan Initiative for World-leading Vaccine Research and Development Centers, Hokkaido University, Institute for Vaccine Research and Development, Hokkaido, Japan; ^6^ International Collaboration Unit, International Institute for Zoonosis Control, Hokkaido University, Sapporo, Hokkaido, Japan; ^7^ Endocrinology Department, Wexford General Hospital, Wexford, Ireland; ^8^ Department of Infectious Diseases, Mater Misericordiae University Hospital, Dublin, Ireland; ^9^ Department of Infectious Diseases, Beaumont Hospital, Beaumont, Dublin, Ireland; ^10^ Department of International Health and Tropical Medicine, Royal College of Surgeons in Ireland, Dublin, Ireland; ^11^ Department of Infectious Diseases, Cork University Hospital, Cork, Ireland

**Keywords:** COVID-19, SARS-CoV-2, mast cells, interferon, gene expression, plasma biomarkers

## Abstract

**Background:**

Dysregulated immune responses to severe acute respiratory syndrome coronavirus 2 (SARS-CoV-2) infection are thought to underlie the progression of coronavirus disease 2019 (COVID-19) to severe disease. We sought to determine whether early host immune-related gene expression could predict clinical progression to severe disease.

**Methods:**

We analysed the expression of 579 immunological genes in peripheral blood mononuclear cells taken early after symptom onset using the NanoString nCounter and compared SARS-CoV-2 negative controls with SARS-CoV-2 positive subjects with mild (SARS+ Mild) and Moderate/Severe disease to evaluate disease outcomes. Biobanked plasma samples were also assessed for type I (IFN-α2a and IFN-β), type II (IFN-γ) and type III (IFN-λ1) interferons (IFNs) as well as 10 additional cytokines using multiplex immunoassays.

**Results:**

We identified 19 significantly deregulated genes in 62 SARS-CoV-2 positive subject samples within 5 days of symptom onset and 58 SARS-CoV-2 negative controls and found that type I interferon (IFN) signalling (MX1, IRF7, IFITM1, IFI35, STAT2, IRF4, PML, BST2, STAT1) and genes encoding proinflammatory cytokines (TNF, TNFSF4, PTGS2 and IL1B) were upregulated in both SARS+ groups. Moreover, we found that FCER1, involved in mast cell activation, was upregulated in the SARS+ Mild group but significantly downregulated in the SARS+ Moderate/Severe group. In both SARS+ groups we discovered elevated interferon type I IFN-α2a, type II IFN and type III IFN λ1 plasma levels together with higher IL-10 and IL-6. These results indicate that those with moderate or severe disease are characterised by deficiencies in a mast cell response together with IFN hyper-responsiveness, suggesting that early host antiviral immune responses could be a cause and not a consequence of severe COVID-19.

**Conclusions:**

This study suggests that early host immune responses linking defects in mast cell activation with host interferon responses correlates with more severe outcomes in COVID-19. Further characterisation of this pathway could help inform better treatment for vulnerable individuals.

## Introduction

Severe acute respiratory syndrome coronavirus 2 (SARS-coV-2) infection, causing Coronavirus Disease 2019 (COVID-19) disease can manifest with diverse clinical presentations. While the majority of infected individuals experience asymptomatic or mild infection, a significant proportion develop severe manifestations such as acute respiratory distress syndrome (ARDS), multi-organ failure, and death (1). Although the global burden from SARS-CoV-2 has declined with vaccination, progression to severe and critical COVID-19 is known to occur more frequently in unvaccinated, older individuals and those with significant immunosuppression (2, 3).

The first line of antiviral defence comprises the innate immune system which is essential for appropriate immune response to viral infection. In the case of infection with SARS-CoV-2, the innate immune system recognises viral RNAs and pathogen-associated molecular pattern molecules (PAMPs) through three main classes of cytoplasmic pattern recognition receptors (PRR): Toll-like receptors (TLRs), RIG-I-like receptors (RLRs) and NOD-like receptors (NLRs), which trigger the expression of interferons (IFN) and activation of antiviral effectors such as natural killer (NK) cells, CD8+ T cells and macrophages (4–8). This cascade of events also leads to stimulation of additional cytokines, such as pro-inflammatory tumor necrosis factor alpha (TNF-a), interleukin-1 (IL-1), IL-6, and IL-18 (5). When operating appropriately, these immune effectors stimulate antiviral responses in targeted cells and drive additional adaptive immune response (9). However, in some individuals with COVID-19, a state of hyper-inflammation and a dysregulated immune response, termed a “cytokine storm” occurs. This is characterised by an inappropriate, over-stimulation of the innate immune response, which contributes to end organ damage, the development of ARDs and is associated with increased mortality (10–12).

IFNs are potent, multifunctional cytokines that have been the focus of much attention in SARS-CoV-2 pathogenesis. Type I and III IFNs in particular play crucial roles in innate immune responses and induction of inflammation during viral infection (13). Type I IFNs include IFN-α, IFN-β, IFN-ω, IFN-, IFN-κ, IFN-δ, and IFN-τ and are produced by all nucleated cell types as their receptor is ubiquitously expressed. Type I IFN to its receptor IFNAR and inducing the expression of the interferon-stimulated genes (ISGs) through the JAK-STAT pathway, inducing an antiviral response (13). ISGs are central to counteracting viral infection and are also implicated in regulating type I IFN induction and downstream signalling. Increased expression of type I IFNs is seen in all stages of COVID-19 disease (14). Significantly, the timing of the IFN response can influnce the course of the disease (15). In a well-regulated immune response, early IFN induction can result in rapid viral clearance and asymptomatic or mild disease. In contrast a deregulated immune response leads to overactivation of this antiviral pathway and inappropriate pro-inflammatory cytokine over-production by innate immune cells, resulting in excessive inflammation, tissue damage and more severe clinical disease (16).

The severity of COVID-19 disease varies between individuals, as a number of endogenous and external factors can influence an individual’s type I IFN response. This IFN signature may be adversely affected by host-dependent genetic and immunological factors. Gene expression analyses of cells is beginning to unravel these host immune dynamics and IFN gene responses (17–19). What remains unclear is which upstream pathways regulate downstream inflammatory effects that determine appropriate or inappropriate type I IFN responses and how these host gene expression patterns vary among individuals with mild, moderate, or severe clinical outcomes. Understanding this crosstalk between early host innate immunity and SARS-CoV-2 disease severity may help explain the imbalance observed in different individual inflammatory responses seen in COVID-19. In the present study, we aimed to determine whether immune-related gene expression early in infection could predict progression to severe COVID-19 disease.

## Methods

### Study design and participants

The All-Ireland Infectious Diseases (AIID) Cohort study is a prospective, multicentre cohort enrolling individuals attending clinical services for infectious diseases in Ireland. Consenting subjects provide data on demographics (age, sex, ethnicity, smoking status), clinical characteristics (hospitalisation, date of symptom onset, and underlying conditions, such as diabetes, malignancies, etc.), and laboratory results. Biological samples are collected and stored for biobanking. For this analysis, we included AIID Cohort participants who presented to the Mater Misericordiae University Hospital and St. Vincent’s University Hospital between April and July 2020 within 5 days of symptom onset for laboratory- confirmed COVID-19. Blood samples were collected and separated into ethylenediamintetraacetic acid (EDTA) whole blood derived plasma frozen at -80°C and peripheral blood mononuclear cells (PBMCs) cryopreserved in liquid nitrogen. Also, SARS-CoV-2 PCR negative controls (SARS-) presenting with respiratory infection matched on age and sex were included. Maximum COVID-19 disease severity attained as well as clinical outcomes (including mortality) was defined according to the WHO guidance and categorised into two groups; SARS+ Mild, and SARS+ Moderate/Severe for analysis (20).

### Patient consent statement

Written, informed consent was provided by all participating subjects. The St Vincent’s Hospital Group Research Ethics Committee and the National Research Ethics Committee for COVID-19 in Ireland reviewed and approved the AIID cohort study in line with national and European regulations on health research.

### NanoString gene expression

The expression of 579 immunological genes were analysed in PBMC using the NanoString nCounter Immunology panels, NS Immunology v2C2328 in the nCounter Analysis System (NanoString, Seattle, USA). Total mRNA was extracted from 2x10^6^ human PBMC using RNeasy^®^ Plus Kit (Qiagen, Germany) and the quality of RNA was assessed on the Agilent RNA 6000 bioanalyser (Agilent Technologies, Germany) according to manufacturer’s instructions. We analysed 200 ng of total RNA from each sample as follows: the RNA was hybridised with the code set of the Immunology panel overnight in batches of 12 samples; the hybridised samples were then prepared in the nCounter Prep Station (NanoString, Seattle, USA), and afterwards the expression of each gene counted with the nCounter Digital Analyzer (NanoString, Seattle, USA) by counting the number of hybridised molecules.

The gene expression from the raw counts were normalised for each sample. The NanoString code set panel contains eight negative probes and six serial concentrations of positive control probes used for quality control of the raw data and for further scaling for comparison during the analysis: the initial step of normalisation for variation associated with the technical platform (batch effect) was carried out using the internal positive controls by calculating the geometric mean of internal positive controls for each sample. Following this, a scaling factor per sample was determined as the ratio of the average across all geometric means and the geometric mean of the sample. For each sample, all gene counts were then multiplied by the corresponding scaling factor. Then, the background level for each sample was calculated as the median of counts + 2 standard deviations across the eight negative probe counts. The background level was subtracted from each gene for each sample. Finally, normalisation for differences in RNA input used the same method as in the positive control normalisation, but geometric means were calculated over 15 housekeeping genes chosen from the forty candidate reference genes provided by NanoString (21).

### Measurement of inflammatory biomarkers

Levels of type I (IFN-α2a and IFN-β), type II (IFN-γ) and type III (IFN-λ1) interferons, interleukins (IL-1 beta, IL-2, IL-4, IL-5, IL-6, IL-10, Il-12p70, IL-13, IL-17A) and TNF-alpha, were measured in biobanked plasma, using custom multiplex electrochemiluminescence immunoassays on the Meso Scale Discovery Platform (MSD, Rockland, USA), which employs electrochemiluminescence for analyte detection. Assays were performed following the manufacturer’s instructions and recommended dilutions for human plasma.

To avoid the effect of freeze-thaw cycles single-use plasma aliquots were used for each biomarker. Samples were processed with controls and standards on each plate along and were performed in duplicate. To ensure quality control, the coefficient of variation (CV) was used. For any samples with an intra-plate CV above 10% and inter-plate CV above 15%, we referenced against controls and standards and repeated if necessary. Any subject that had a biomarker measurement with CVs above the previously specified thresholds after 2 to 3 repeats was identified as having an unreliable biomarker measurement and removed from subsequent analyses.

### Statistical analysis

For clinical factors, categorical and continuous variables were summarised using frequency/percentage and median with interquartile range (IQR), respectively. Comparison of categorical variables were performed using a Chi-squared analysis and Kruskal- Wallis test. The SARS-CoV-2 positive subjects were stratified into two groups according to WHO-classified disease severity; the mild group (SARS+ Mild) and the moderate/severe group (SARS+ Moderate/Severe). We analysed the changes in gene expression between these groups against the SARS-CoV-2 negative (SARS -) subjects. Genes were considered to be significantly deregulated if the log_2_ fold change (FC) in either or both the SARS+ groups were significantly different by at least 0.5 (log_2_ FC > 0.5) setting a statistical significance threshold on *p* < 0.05 and applying a Benjamini-Yekutieli *p*-value adjustment to correct for the false discovery rate. The criteria for significantly deregulated genes was independently applied in the two comparisons of the SARS+ Mild vs SARS - and in SARS+ Moderate/Severe vs SARS -. We used Kruskal–Wallis tests to examine differences in biomarker levels between COVID-19 severity groups. All statistical tests were two-tailed at the significance level of *p <*0.05.

## Results

### Subject characteristics

Between April and July 2020, we recruited 115 subjects, 61 subjects with PCR-confirmed COVID-19 (SARS+) and 54 SARS - negative controls. Within the SARS+ group, most (52%) experienced only mild disease, 23% moderate, 11% severe disease and 14% critical-ARDs, respectively. The median age of the SARS+ and SARS - groups were similar (64.8 [50–74] years and 64.5 (49.25-78.75) years, respectively). Among both study groups, males and those of Caucasian ethnicity were the most represented (See **Table 1** for subject demographics). Compared with the SARS - group, subjects in the SARS + groups were more frequently diabetic, likely to have hypertension, likely to be obese and less likely to have renal disease. SARS-CoV-2 cycle threshold (Ct) values were available for 44 of the 61 with PCR-confirmed COVID-19 (SARS+) subjects. We observed no association between Ct value and COVID-19 disease severity.

**Table 1:** Characteristics of the Study Population.

	SARS + Mild	SARS + Mod/Sev	SARS -	p-value
Characteristic	N= 32	N = 29	N= 54	
**Age**	62.2 (50,84)	66 (56,72)	64.5 (49.25-78.75)	>0.9
**Gender, Male, No (%)**	19 (59)	20 (569)	35 (65)	0.4
**Ethnicity, n (%)**				0.89
*Caucasian*	26 (81)	18 (62)	41 (76)	
*Asian*	0	3(10)	2 (3.7)	
*Black*	2(6)	1(3)	0 (4)	
*Other*	3(9.4)	1(3.4)	4 (7.4)	
**BMI (kg/m^2^)**	25(23.4, 29)	28.6 (26.65,33.7)	25 (22-28)	0.021
**Comorbidity present, n (%)**	27 (84)	22 (76)	39 (74)	0.5
*Hypertension*	14 (44)	14 (48)	19 (35)	0.5
*Diabetes*	3 (9)	6 (21)	5 (9.3)	0.3
*Respiratory disease*	5 (16)	9(31)	10 (54)	0.3
*Heart disease*	9 (28)	5(17)	16 (30)	0.4
*Renal Disease*	2(6)	1(3)	7 (13)	0.4
*Liver Disease*	0	1(3)	1 (2)	0.7
*Obesity*	4 (12)	8 (28)	4 (7.4)	0.043
*Malignancy*	5(16)	7(24)	12 (22)	0.7
*Immunosuppressive disease*	2(6)	1(3)	3 (5.6)	>0.9
*Neurological disease*	3 (9.4)	0	7 (13)	0.12
*Other*	17 (53)	12(41)	26 (48)	0.7
**Medical Treatment**				
*antibiotics, n (%)*	16 (50)	17 (59)	30 (56)	0.5
*antivirals, n (%)*	5 (16)	11(38)	1 (2.2)	<0.001
*immunosuppressant, n (%)*	2 (6)	6 (21)	8 (15)	0.68
**SARS-CoV-2 Replication levels,** **Cycle Threhold (Ct)**	N= 25	N=19	NA	0.3
	23.04 (18.83, 30.31)	29 (22.36, 31.02)		

Data are median (IQR) unless otherwise stated. COVID-19, coronavirus disease 2019; SARS-CoV-2, severe acute respiratory syndrome coronavirus 2; WHO, World Health Organization. Disease severity assigned according to WHO criteria (22). NA, Not Applicable.

### Differential expression of genes between COVID-19 cases and uninfected controls

Analysis of the NanoString gene expression identified a total of 19 significantly deregulated genes in the SARS+ groups (**Table 2**). Among these deregulated genes, five related to cytokine signalling, nine to type I interferon signalling, while a further nine genes mapped to innate immune functions.

**Table 2:** Deregulated gene expressions SARS + compared to SARS -.

Deregulated genes	Type I interferon Signalling	Cytokine Signalling	Innate Immune Function	TNF Signalling
*MX1*	↑	↑	↑	
*STAT1*	↑	↑		
*STAT2*	↑	↑		
*BST2*	↑		↑	
*IRF7*	↑		↑	
*PML*	↑			↑
*IFI35*	↑			
*IFITM1*	↑			
*IRF4*	↑			
*IL1B*		↓		
*SRC*		↑		
*FCER1A*			↓	
*ICAM2*			↑	
*ICAM4*			↓	
*IFI16*			↑	
*PTK2*			↑	
*SERPING1*			↑	
*TNFRSF17*				↑
*TNFRSF4*				↑

**Figure 1:**
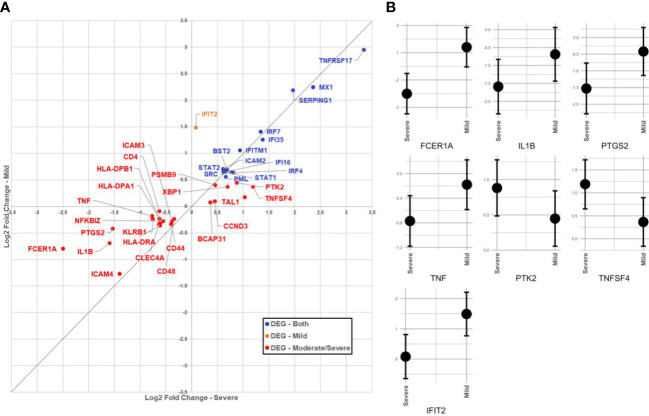
**(A)** Comparison of immunological gene expression changes/severe and Mid COVID-19 cases. The horizontal and vertical axes show the logarithmic fold change against negative sample in moderate/severe and mid-cases, respectively. The identity line has been added as guide for genes with similar changes. Genes have colored according to the significance of expression change in both (blue), only in the mid moderate/severe group (red). **(B)** Comparison of confidence intervals of log2 fold changes in between moderate/severe and mild COVID-19 cases. The vertical axes of panels show the confidence interval of the fold-change and the horizontal axes show the groups. Displayed transcripts are FCER1A, IL1B, TNF, PTGS2, IFIT2, TNFSF4 and PTK2.

In the SARS+ groups, those with Moderate/Severe disease showed greater downregulation of *FCER1A* than those with mild disease (see **Table 2**; **Figure 1**). This gene encodes the high-affinity IgE receptor, also known as FcϵRI found on mast cells and is activated upon IgE binding. Mast cells produce various pre-formed and newly synthesised mediators upon activation with IgE, including TNF alpha, prostaglandins and IL-1 β. Consistent with this, we also found corresponding downregulation of the *TNF, PTGS2* and *IL1B* genes in the Moderate/Severe group (**Figure 1A**) suggesting downregulation of mast cell activation accompanying downregulation of FcϵRI expression.

In the SARS+ groups, we found upregulation of genes involved in IFN signalling, including *MX1, IFI6, STAT1/2, IRF4* and *IRF-7*. The *MX1* gene encodes a guanosine triphosphate (GTP)-metabolizing protein that is involved in the cellular antiviral responses. The encoded protein is induced by type I and type II interferons and antagonises the replication process of several different RNA and DNA viruses (23). Interestingly, those with Mild disease also had greater upregulation of *IFIT2*, an ISG belonging to the IFN-induced protein with tetratricopeptide repeats (IFIT) family. This gene encodes a family of proteins that are induced after viral infection, or PAMPs recognition (24) (**Figure 1B**).

We found deregulation of several genes related to innate immune function, including relative upregulation of *SERPING1* and *ICAM2* genes in the SARS+ groups. *SERPING1* encodes for the C1 inhibitor protein, a regulatory component of the complement cascade, and *ICAM2* enables adhesive interactions necessary for antigen-specific immune responses, NK-cell mediated clearance and the recirculation of lymphocytes. In those with Moderate/Severe disease we observed greater downregulation of *HLA-DRA, HLA-DPB1* and *HLADPA1* compared to those with Mild disease and compared to the SARS- controls. This suggests an imbalanced activation of HLA class I and II antigen presentation, consistent with over-activation of innate immune responses.

Other dysregulated genes in the SARS+ group included *TNFRS17*, involved in TNF function, which was markedly upregulated. *TNFRS17* is expressed in mature B lymphocytes and encodes a member of the TNF-receptor superfamily. It plays a role in MAPK8/JNK and NF-kB activation pathways and enables the development and survival of B-cells to sustain humoral immunity. Similarly, we observed upregulation of *TNFSF4* in those with Moderate/Severe disease. This gene encodes for an OXO ligand which activates CD4+ cells to initiate adaptive T-cell responses in allergic inflammation. This gene has been associated with various autoimmune and inflammatory conditions (25).

### Plasma Interferon levels

**Figure 2:**
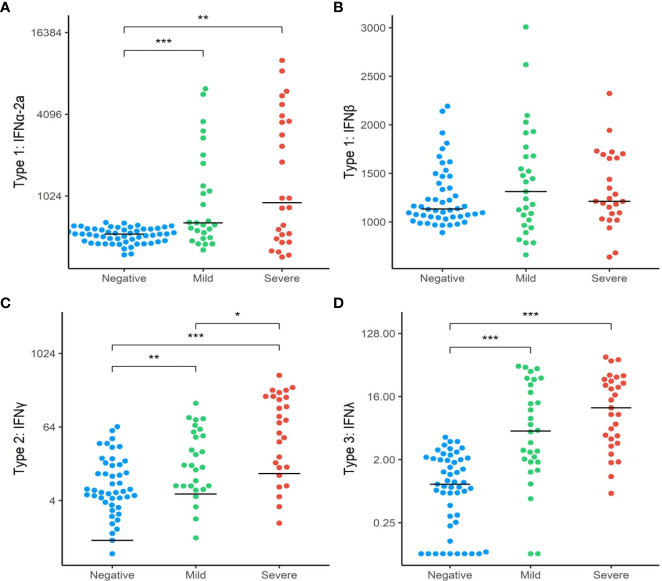
Interferon protein levels by COVID-19 disease severity. The distribution of interferon protien levels **(A)** Type 1 interferon-α2a, **(B)** Type 1 interferon-β, **(C)** Type 2 interferon-γ and **(D)** Type 3 interferon-λ in SAR-CoV-2 Mild and Moderate/Severe groups were compared to patients in the SARS- group. Only significant comparisons are shown: *p<.05, **p<.01, ***p<.001. IFN, Interferon. Bars represent median values shown.

To further explore whether the differences found in the interferon gene expressions between the three groups translated into functional differences in protein expression, we analysed downstream targets of these genes through analysis of plasma biomarkers. We first compared the relative concentrations of type I interferon subtypes (IFN1a + IFN1b) between the SARS+ and SARS- groups. Circulating levels of IFN2a, IFNγ and IFNΛ1 were higher in the SARS+ group compared to the SARS- group (**Figure 2**). However only IFNγ was different between the SARS+ disease severity groups, with significantly higher IFNγ levels observed in the Moderate/Severe group compared to the Mild group (**Figure 2C**).

### Plasma cytokine levels

**Figure 3:**
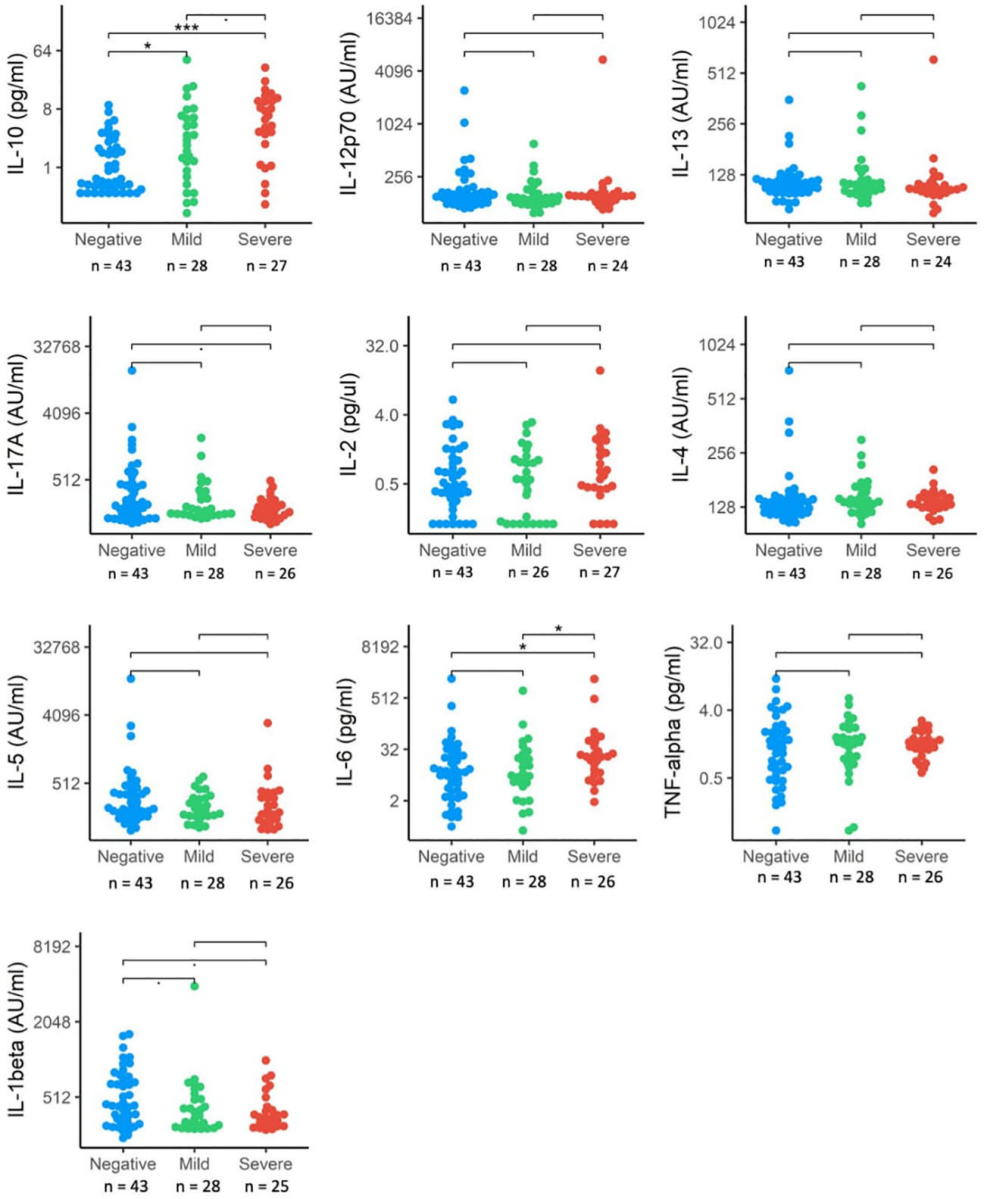
Interleukin protein levels by COVID-19 disease severity. The distribution of interleukin expression (IL-10, IL-12p70, IL-13, IL17-A, IL-2, IL-4, IL-5, IL-6, TNF-alpha, IL-beta) in SRS-Cov-2 mild and severe disease severity groups compared to SASRS-Cov-2 negative patients. NS, no significant. *p<.05, ***p<.001.

We also investigated differences in cytokine signaling pathways between these groups, by direct detection of circulating cytokines induced by IFN responses following SARS-CoV-2 infection. These included interleukins (IL-1 β, IL-2, IL-4, IL-5, IL-6, IL-10, Il-12p70, IL-13, IL-17A) and TNF-α (**Figure 3**). Circulating levels of IL-1 β, IL-6, IL-17A and IL-10 were significantly elevated in the SARS+ group compared to the SARS- group. When comparing the Moderate/severe to Mild disease groups, we further observed increased levels of IL-6, a pro-inflammatory cytokine and known marker for severe COVID-19 disease and increased levels of IL10, an anti-inflammatory cytokine. We found no changes however in the other markers of inflammation (IL-1 β, IL-17A and TNF-α) and no changes in markers of T-cell differentiation, stimulation, or regulation (IL-12p70, IL-2, IL-4, IL-5, IL-13).

IL-10, together with IL-4 is part of an auto-regulatory feedback loop on mast cells. Activation of mast cells found in the respiratory tract by SARS-CoV-2 can be seen in the initial stage of infection and through stimulation of the FcϵRI, mast cell activation leads to the release of inflammatory cytokines and chemokines (26). This suggests a link between the down-regulated FcϵR gene expression and upregulated IL-10 concentrations seen in those with Moderate/Severe disease.

To explore relationships between gene expression and downstream functional pathways, we assessed correlation analyses between the expression of FCER1A and the biomarker levels (cytokines and interferon levels) and considered the disease outcome. We found a number of statistically significant associations, which further suggest that the signatures we have observed are true biological signatures. In all SARS-CoV-2 cases, FCER1A expression positively correlated with IL-16 and IL-23A and negatively correlated with IFN-α2a. In mild cases, FCER1A was significantly positively correlated with the expression of interferon gamma, IL-27, IL18, IL-10 and IL-1 beta and negatively correlated with circulating levels of IL-2 and TNF-alpha. In contrast, in moderate/severe disease we observed positive correlations between FECR1A expression and circulating levels of IL-1 beta, IL-5 and IL-13. These associations were unchanged when accounting for Ct values and time of symptom onset.

## Discussion

The balance between activation and suppression of innate immunity is essential for adequate host immune responses to respiratory viral infections and helps to prevent immunopathological end organ effects from uncontrolled inflammation such as ARDs. Our analysis demonstrates a novel link between the expression of a gene important for mast cell function and IFN signatures in PBMCs with downstream systematic mediators of antiviral and inflammatory responses. Together, these interactions can influence clinical outcomes in acute SARS-CoV-2 infection.

**Figure 4:**
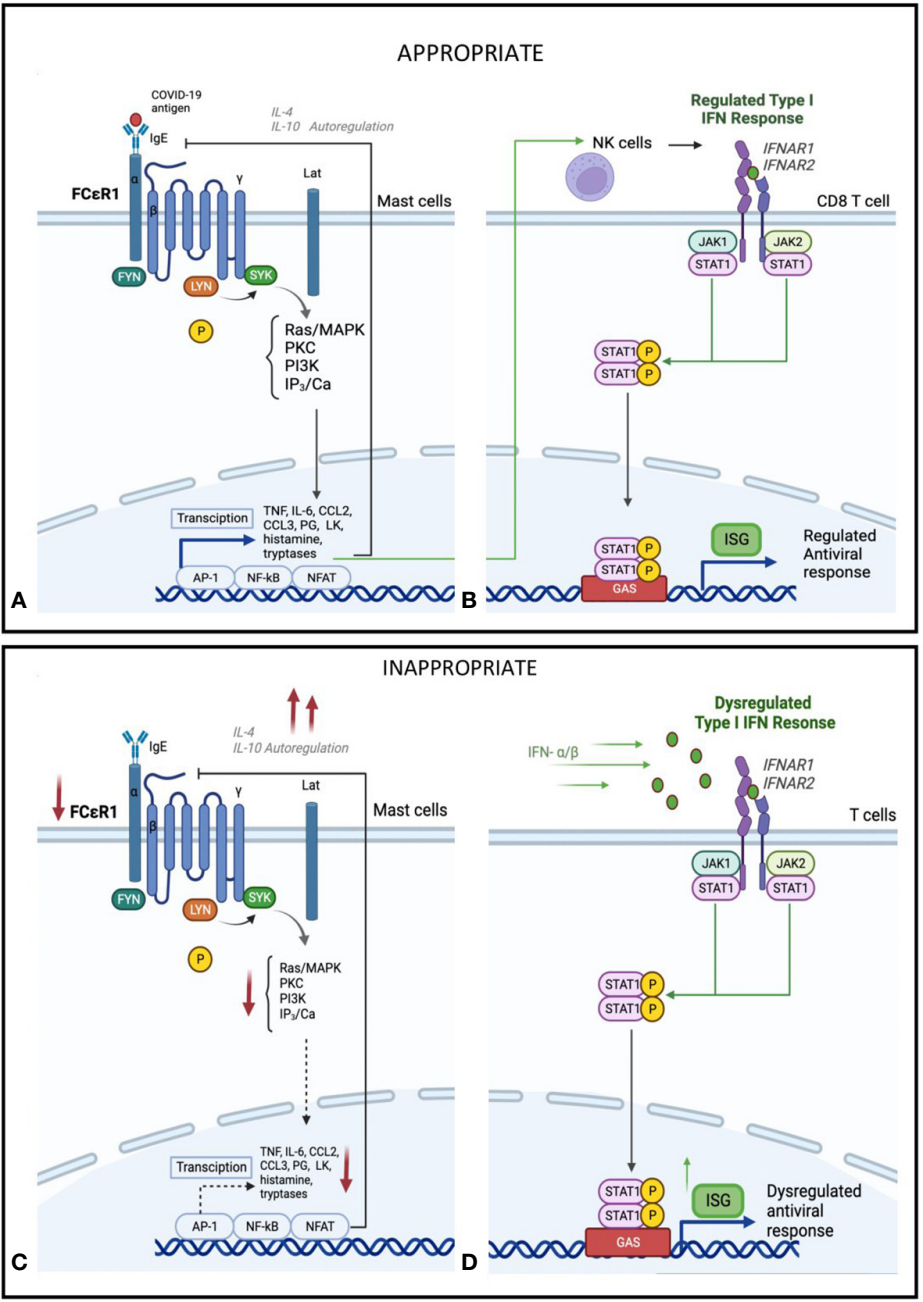
Hypothesis of proposed **(A)** Mast cell activation and **(B)** IFN-I regulation in early or mild SARS-CoV-2 infection. Mild Sars-CoV-2 infection activates Immunoglobulin E (IgE) receptors on mast celld through FcεR1, the high-affinity receptor for the Fc region of IgE. This triggers an intracellular signalling cascade, leading to de novo production of cytokines and lipid mediators, including TNF, IL-6, CCL2, CCL3, prostaglandin (PG) D2 anf E2, and leukotriene (LK) B4 and C4. This acute inflammatory response stimulates NK cell activation and type I IFN (IFN-α), activation. This leads to induced expression of interferon-stimulated genes (ISGs) via the JAK-STAT signalling pathway and a regulated inflammatory mediator production. Feedback control through IL-4 and IL-10 stimulated autoregulation attenuates a controlled anti-viral response. Hypothesis of proposed **(C)** Mast cell suppression and **(D)** IFN-I dysregulation in severe SARS-CoV-2 infection. In severe SARS-CoV-2 disease, in addition to mast cells, other immune cells, particularly Toll-like receptor-expressing T-cells (TLR) 3, 7, and 8, recognises the virus using pathogen recognition receptors (PRRs), attenuating interferon production. In addition, IFNs are secreted in an autocrine and paracrine manner to induce expression of ISGs. This dysregulated phase of SARS-CoV-2 disease sees an amplified innate immunity and high levels of proinflammatory cytokines that results in a sustained cytokine/ chemokine release, termed a "cytokine storm" (28). Reduced IgE activation of FcεR1 by virus, leading to a decrease in mast-cell activation, as well as negative feedback from IL-4 and IL-10, leading to a decrease in overall mast cell activity (29, 30).

We found greater downregulation of FcεR1 gene expression in those with moderate/severe COVID-19 suggesting derangement in this innate immune response early in the course of COVID-19 (**Figure 4**). Mast cells act as “first responders” and rapidly secrete preformed mediators including tryptase, granule-stored heparin, tryptase and TNF as well as newly synthesised leukotrienes, platelet-activating factor, prostaglandins, cytokines and chemokines (27, 28). Mast cells are innate immune cells that also participate in the adaptive immune response to viral infections (29). Although found ubiquitously in the body, mast cells usually concentrate in the respiratory tract and skin. Following stimulation by various cytokines, mast cell progenitors migrate to and differentiate in the target tissue. Under normal conditions, mature mast cells do not circulate in the bloodstream but can line blood and lymphatic endothelium and have been implicated in dengue-virus-associated vasculopathy (30). Upon viral infection, stimulation occurs through allergen crosslinking allergen-specific immunoglobulin E (IgE) bound to high-affinity FcϵRI (10). Appropriate mast cell activation leads to a protective immune response, by direct activation against viral infection and stimulating the immune system. However, early markedly downregulated FcεR1 gene expression observed in Moderate/Severe COVID-19 suggests an inappropriate response, which is further supported by the down regulated targets such as TNF, PTGS2 and IL-1B. These findings are supported by results from a genome-wide gene expression profiling of human mast cells stimulated by IgE or FcϵRI-aggregation (31). This study found an association between these genes and downstream function gene targets of cytokine and chemokine expression, including IL-1β, several genes encoding for transcription factors and multiple members of the TNF family. Together, these findings suggest that a reduced expression of FcεR1 dose impact on the function of relevant downstream antiviral immune responses.

Our findings contrast with previous studies which found an association between increased mast cell activation and severe COVID-19 (26, 32–34). However, these studies focused on localised mast cell responses in lung or alveolar tissue in individuals with severe COVID-19 rather than transcriptional regulation of mast cell function early in infection. Interestingly, a recent study in mast cell-deficient mice showed that that a lack of mast cells may lead to increased viral uptake of upper respiratory tract cells, leading to development of more severe clinical disease. Furthermore, these mast cell-deficient mice also exhibited persistent infection in the post-acute setting (35). Together with the interpretation of our findings, these studies suggest that a defect in mast cell responses may contribute to severe COVID-19 disease.

Mast cells are an important and arguably underappreciated local source of type I and III interferons following viral infection (36). Mast cells contribute to host antiviral responses by recruiting CD8+ T cells, which produce type I IFNs such as IFN-α and IFN-β. We found consistent deficiencies in transcriptional signatures of IFN-related genes together with IFN-induced responses in this study. IFN responses are important drivers of timely resolution of infection in viral diseases (37). The predominant anti-viral IFN class is Type I IFN, consisting of IFNα and IFNβ subtypes. Despite the roles of type I interferons in viral clearance, there remains ambiguity regarding its role in COVID-19 (37). An increasing body of evidence suggests a sustained, dysregulated type I IFN response in severe COVID-19 that contributes to poorer outcomes (38, 39). Type I IFN is can also suppress mast cell function and histamine release and recently was shown to play a critical role in controlling mast cell homeostasis (40, 41).

Type I, II and III IFNs activate signalling cascades through the IRF and STAT pathways that induce ISG expression. We observed increased expression of ISG in both SARS+ groups, suggesting a greater role in SARS-CoV-2 infection. Furthermore, we observed differential early expression of some ISGs between those who developed mild versus moderate/severe COVID-19. In particular, *IFIT2*, which is induced rapidly after viral infection and alters proinflammatory cytokine responses was preferably upregulated in those with mild COVID-19 (42, 43). Overexpression of *IFIT2* has been shown to block SARS-CoV-2 infection *in vitro (* 44). This would be consistent with a protective effect of upregulated IFIT2 expression in those who developed mild COVID-19 which was not seen in those with moderate/severe disease (44).

IFN expression is regulated by several intrinsic components and can stimulate as well as inhibit its own expression. IFN signalling can be amplified by the induction of STAT1 and IRF9 expression (45). In contrast, Interferon regulatory factor 7 (IRF7) has emerged as a crucial type I IFN regulator. We found upregulation of both STAT1 and IRF7 genes in both SARS+ groups, consistent with a specific activation of this pathway by SARS-CoV-2. This was further confirmed by increased levels of circulating type I (IFNα), type II (IFNγ) and type III IFN seen in the SARS+ compared to SARS- group (**Figure 2**), with a significantly higher IFNγ in those with moderate/severe COVID-19 compared to mild COVID-19. These findings support the hypothesis that a dysregulated IFN response contributes to severe COVID-19 disease. This is further supported by the finding that pre-existing autoantibodies against type I IFNs in those with inborn errors of type I IFN production have been linked severe COVID-19, by inhibiting receptor binding of type I IFNs, impairing type I IFN immunity (31) (46, 47).

Our findings are in keeping with previous gene expression studies examining disease severity in COVID-19. A similarly- sized study of 124 participants with samples collected at two time points, 1-5 days post hospital admission and 6-20 days post admission, found upregulation of several pathways in those with severe COVID-19 disease. These pathways included neutrophil degranulation, interferon α/β/γ signalling, IL-4 and IL-13 signalling, oxidative stress and TNF-α signalling *via* NFκ-B a (48). They also compared the timing of sampling with disease outcome and although gene expression trajectories change over two time points taken, they found that the signatures were still significantly dysregulated between severe versus moderate COVID-19 and between those who died and those who survived. A smaller study conducted in the ICU setting in those with severe disease (n= 31) also found enrichment of IFN and neutrophil gene signatures (49). Interestingly, they also found no significant differences in viral loads between the COVID-19 patient groups despite their clear differences in clinical features and gene expression profiles.

The findings in these relatively small studies are supported by a larger whole-genome sequencing study of 7,491 critically ill covid-19 subjects, where they found 23 replicated genetic associations with critical COVID-19 (50). Of these, 5 variant genes have direct roles in interferon signalling (*IL10RB* and *PLSCR1*) as well as genes involved in coagulation (*SELE*, *ICAM5* and *CD209).* A link between coagulation pathways and severe COVID-19 disease has consistently been found in other studies of gene expression (51, 52). We also found upregulation of several genes involved in platelet adhesion including ICAM2, ICAM 4 and PTK2 supporting these studies. In addition to coagulation pathways, upregulation of genes involved in complement activation and dysregulated lipid transport are seen in those with severe disease (53). It is evident from these gene expression studies that several biological pathways are involved in COVID-19 pathogenesis. Unlike these studies, our findings revealed an association between dysregulation of a mast- cell specific gene and antiviral responses. Further work exploring these interactions may unveil an important role for mast cell function in COVID-19.

Taken together, our findings suggest a hypothesis that gene expression changes in mild COVID-19 stimulates an appropriate inflammatory response, involving mast cell activation that can drive downstream expression of cytokines and IFN mediators (**Figures 4A, B**). Yet in those with severe disease, we see a defect in this mast cell specific gene and subsequent downstream expression of ISGs combined with a dysregulated inflammatory response, characterised by higher type II IFN and systemic inflammatory cytokines (**Figures 4C, D**). Thus, both deficiencies in mast cell responses and type II IFN hyper-responsiveness could be linked to severe COVID-19, suggesting that early host immune responses could be a cause and not a consequence of severe COVID-19.

Our study has a number of limitations. Although we used a sample size similar to other transcriptomic studies, our findings should be further validated using a similar approach in other independent cohorts. Further functional studies should be performed to validate the severity-related pathways detected. In particular, as our data is derived from total PBMC transcriptional profiling. As such, we cannot out rule that decreases in gene expression may reflect decreases in the total amount of specific cell types within the PBMC pool (such as mast cells) as compared to downregulation of expression within those cells. Further research should focus on exploring and validating these function pathways directly within mast cells. Although this study was a cross-sectional analysis, which limits the ability to determine causal relationships, the timing of the sampling predated assessment of final, subsequent COVID-19 disease severity. Although the sampling was early in disease course, we cannot rule out different transcriptional changes occurring even earlier after acquisition of SARS-CoV-2. Of the available viral load measurements, we did not find a significant difference between disease severity groups. One caveat to this is that our viral load measurements were taken from nasal swabs, and it is possible that increased viral presence in the lower airway may lead to worse disease outcomes. Furthermore, as the subjects were sampled early in the pandemic and prior to the introduction of vaccines, the impact of vaccination and/or emergence of new SARS-CoV-2 variants on host immune responses was not assessed. Nevertheless, predictors of early markers of inflammation that could identify those most at risk of developing severe disease despite vaccination status will remain an invaluable tool for disease diagnostics.

Despite these limitations, this study shows that early transcriptional pathway assessment of host immune responses links a mast cell-specific gene and differences in downstream innate immune and host antiviral interferon responses with disease outcomes in COVID-19. Further work is required to determine the underlying pathophysiological mechanisms behind potential mast cell interactions and their potential to act as future, therapeutic targets.

## Conclusions

In summary, this study identified a number of differentially-expressed genes that distinguished those with severe COVID-19 disease from those with mild or moderate illness. These transcriptional signatures identified dysregulated genes involving interferon and innate immune pathways as well as a mast cell-specific gene together with downstream corresponding circulating cytokine and interferon responses. Future prospective studies are needed to explore these mechanisms further and to determine the suitability of these discriminatory pathways as potential therapeutic targets.

## Data availability statement

The datasets presented in this study can be found in online repositories. The name of the repository and accession number can be found here: GSE227080 (GEO).

## Ethics statement

The studies involving human participants were reviewed and approved by St. Vincent’s Hospital Group Research Ethics Committee and the National Research Ethics Committee for COVID-19 in Ireland. The patients/participants provided their written informed consent to participate in this study.

## Author contributions

PM, GG, & RMC conceived and designed the study. MC and RMC obtained funding. GG, RMC and AA analyzed and interpreted the data. RMC contributed to data collection. RMC drafted the manuscript. RMC, PM, MC, GG and AA revised the manuscript. All authors contributed to the article and approved the submitted version.

## All-Ireland Infectious Diseases (AIID) cohort study investigators

Mater Misericordiae University Hospital: A. Cotter, E. Muldoon, G. Sheehan, T. McGinty, J. S. Lambert, S. Green, K. Leamy. St Vincent’s University Hospital: G. Kenny, K. McCann, R. MacCann, C. O’Broin, S. Waqas, S. Savinelli, E. Feeney, P.W. G.Mallon. Centre for Experimental Pathogen Host Research: A. Garcia Leon, S. Miles, D. Alalwan, R. Negi. Beaumont Hospital: E. de Barra, S. McConkey, K. Hurley, I. Sulaiman. University College Cork: M. Horgan, C. Sadlier, J. Eustace. University College Dublin: C. Kelly, T. Bracken. Sligo University Hospital: B. Whelan. Our Lady of Lourdes Hospital: J. Low. Wexford General Hospital: O. Yousif. University Hospital Galway: B. McNicholas. St Luke’s Hospital Kilkenny: G. Courtney. Children’s Health Ireland: P. Gavin.
